# Preparation and Chemical/Microstructural Characterization of Azacrown Ether-Crosslinked Chitosan Films

**DOI:** 10.3390/ma10040400

**Published:** 2017-04-11

**Authors:** Julius Toeri, Anayancy Osorio-Madrazo, Marie-Pierre Laborie

**Affiliations:** 1 Freiburg Materials Research Center, University of Freiburg, Stefan-Meier-Straße 21, 79104 Freiburg, Germany; anayancy.osorio@imtek.uni-freiburg.de (A.O.-M.); marie-pierre.laborie@biomat.uni-freiburg.de (M.-P.L.); 2 Forest Biomaterials, University of Freiburg, Werthmanstr. 6, 79085 Freiburg, Germany; 3 Laboratory of Sensors, Institute of Microsystems Engineering IMTEK, University of Freiburg, Georges-Köhler-Allee 103, 79110 Freiburg, Germany

**Keywords:** azacrown ether, chitosan crosslinking, chitosan films, synchrotron X-ray diffraction, adsorption

## Abstract

Chemically stable porous azacrown ether-crosslinked chitosan films were prepared by reacting varying molar amounts of N,N-diallyl-7,16-diaza-1,4,10,13-tetraoxa-dibenzo-18-crown-6 (molar equivalents ranging from 0, 0.125, 0.167, 0.25 and 0.5) with chitosan. Their chemical and structural properties were characterized by solid state-nuclear magnetic resonance (NMR), elemental, Fourier transform infrared (FTIR), microscopy, and X-ray analyses, as well as gel content. NMR and FTIR analyses of the reaction products suggested that new –CH_2_– crosslink bridges were produced between the amine groups of chitosan (Ch) and the allyl groups of the azacrown (DAC). The crosslinking chemistry between allyl and amine groups of the reactants was further evidenced with solution NMR studies on model compound of glucosamine with the azacrown. X-ray diffraction analysis of the Ch/azacrown films using wide angle X-ray scattering (WAXS), including synchrotron-WAXS, revealed that the crystalline arrangement of chitosan (Ch) was partially destroyed with increasing grafting of azacrown ether proportion on the Ch polymer chain. Solubility and gel content determination confirmed network formation with a gel content as high as 84–95 wt %. Microstructural analysis revealed microporous morphology with high surface area. The morphology and structure of the azacrown ether-crosslinked chitosan films could be tailored by stoichiometry of the reacting species.

## 1. Introduction

Crosslinked chitosan, based on various crosslinking chemistries [1,2] has been a strategy of choice to prepare chemically-modified chitosan for water remediation, viz, metal adsorption [3]. The conventional chitosan (Ch) crosslinking agents, including dialdehydes [4,5,6], epichlorohydrin and ethylene glycol diglycidyl ether etc. [7,8,9,10] consume the active amine sites of chitosan during crosslinking and thus may weaken the adsorption efficiency of chitosan. The use of crosslinking agents with functional groups which also serve as metal binding sites can be an effective way to obtain adsorbents with good adsorption capacity.

Among the possible crosslinkers, azacrown ethers are particularly interesting as they are also strong chelators [11,12]. Unlike the conventional crosslinking agents, azacrowns incorporate an electron-rich central cavity with N, O, P or S atoms as part of their active sites [13], thus enhancing interactions with ions through coordinated bonds [14,15]. Furthermore, the overall size of the crown ether cavity might influence the selectivity for various ions [16]. For example, Wan et al. [17] prepared Ch derivatives containing crown ether. The chitosan derivatives had stronger complexation and better and better selectivity for metal ions than corresponding crown ethers and Ch alone. These Ch derivatives could be used to separate and concentrate heavy or precious metal ions in aqueous environments. Other work also reports on the use of chitosan/crown ether derivatives for metal ion adsorption [18,19,20,21,22]. Ding et al. [23] prepared an azacrown ether-crosslinked Ch derivative using N,N-diallyl dibenzo 18-crown-6 crown ether. The group studied its adsorption properties for Pd^2+^, Ag^+^, Pb^2+^, Ni^2+^, Cu^2+^, Cd^2+^, and Co^2+^ and found that it had good adsorption properties for palladium and silver, which was attributed to the presence of the azacrown. Infrared spectroscopy further suggested a chemical reaction between Ch and the crown ether, hinting to the possibility of network formation.

**Scheme 1:**
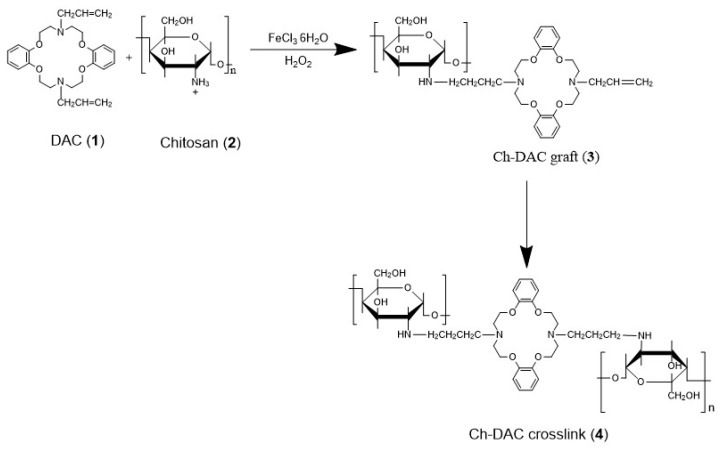
Synthesis of azacrown ether-crosslinked chitosan (Ch-DAC) showing the possible reaction of DAC as a graft or crosslinker of chitosan.

Effective crosslinking of chitosan with azacrown ether is indeed needed to generate a solid film for water remediation. Such film should not disintegrate in water and might thus be easily recovered after use. However, the controlled preparation of stable azacrown ether-crosslinked Ch films (Ch-DAC) has not been reported. Also, the crosslinking chemistry, while expected to arise between the amino group and the allyl of the azacrown, has not been fully elucidated. We hypothesize that the reactants’ stoichiometry can be used to design the composition, structure, crosslinking degree and morphology of azacrown ether-crosslinked Ch film (which in turn should govern its adsorption capacity). We also hypothesize that the crosslinking chemistry involves the hydroamination of the alkene arms of DAC (Scheme 1). The formal addition of the N–H bond of the amine across the alkene C=C double bond is said to yield alkylated amines in a direct, 100% atom-economic process [24,25,26,27,28]. 

In a previous study, we reported improved production of DAC (molecule **1**) with high purity [29]. In the present study, we thus aim to prepare and characterize a series of azacrown ether-crosslinked chitosan (Ch-DAC) films by reacting Ch with N,N-diallyl-7,16-diaza-1,4,10,13-tetraoxa-dibenzo-18-crown-6 ether (DAC) in controlled molar ratios (Scheme 1). Furthermore, we aim to reveal the crosslinking chemistry via model compound studies. 

We thereby demonstrate that the composition, structure and morphology of this series of azacrown ether-crosslinked Ch films can systematically be characterized. The potential to tailor the composition, degree of substitution and morphology of the Ch-based films via stoichiometry is also demonstrated.

## 2. Results and Discussion

### 2.1. Basic Characteristics of the Chitosan Starting Material

Prior to its use, the starting chitosan was characterized for molecular size, degree of acetylation (DA) and morphology. Size-exclusion chromatography (SEC) indicated that it had a weight-(*M*_w_) and number-average (*M*_n_) molecular weights of 1.810 × 10^5^ (±0.30%) g/mol and 8.202 × 10^4^ (±0.57%) g/mol, respectively. The polydispersity index, PDI (*M*_w_/*M*_n_) was found as 2.21 (±0.65%). Solution NMR revealed a DA of 23%. The crystallinity index (CrI) measured by wide-angle X-ray spectroscopy (WAXS) was found to be 75.7%. Water content was about 7% while ash content was 0% at 730 °C, implying that there was no significant inorganic content in the original Ch. Average mass of the monomeric unit (M_0_) of Ch was calculated from the degree of acetylation using the formula; M_0_ = 161.15 + 42DA [30] and was equal to 170.8 g/mol.

### 2.2. Composition and Crosslinking Chemistry of the Azacrown Ether/Chitosan Films

#### 2.2.1. Carbon-NMR Analysis

**Scheme 2:**
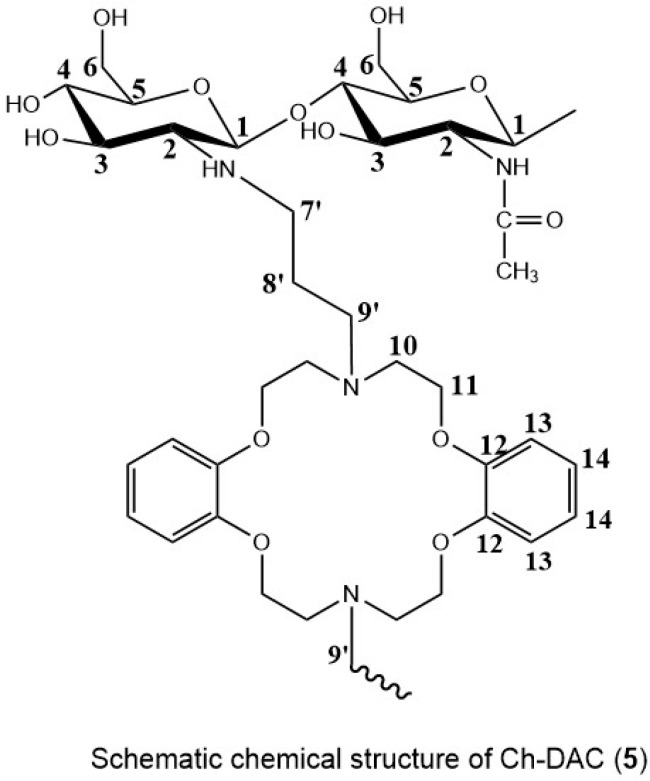
Schematic chemical structure of Ch-DAC (**5**).

**Figure 1:**
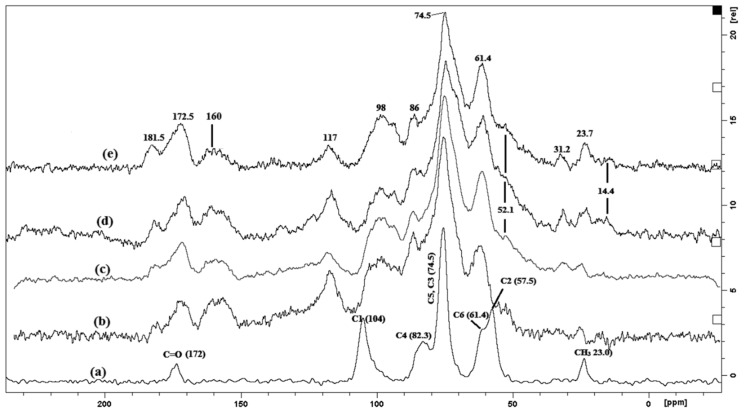
C-NMR spectra of (**a**) natural chitosan and (**b**–**e**) Ch-DAC-modified chitosan with varying DAC content from 0.125 to 0.5 mol/mol chitosan, respectively.

**Figure 2:**
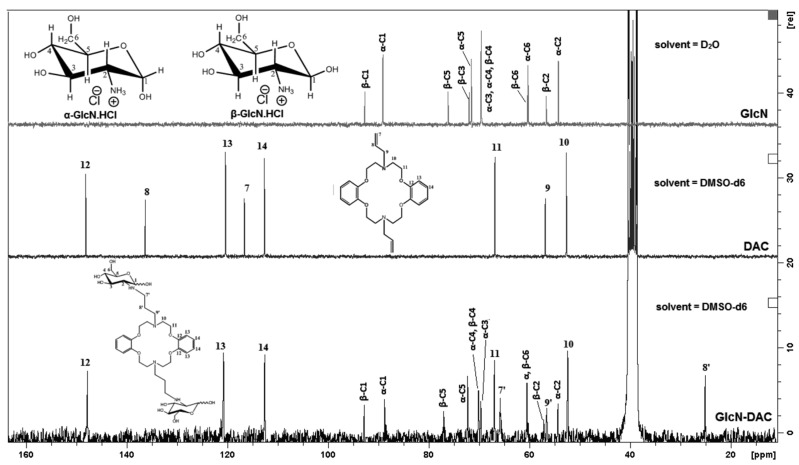
^13^C-NMR spectra of glucosamine, azacrown ether and glucosamine-azacrown ether complex.

Carbon-NMR spectra of natural chitosan and Ch-DAC samples are shown in Figure 1. The labeled peaks in spectrum (a) are the expected series of peaks of the used Ch (see molecule **5**, Scheme 2). They include the peaks at 23.0 and 172.7 ppm associated with the methyl and carbonyl groups of the monomeric form of chitin due to incomplete deacetylation. The other chemical shifts correspond to C1 (104.3 ppm), C4 (82.3 ppm), C5 and C3 (74.5 ppm), C6 (60.6 ppm) and C2 (56.8 ppm). After reaction with DAC, new chemical shifts appear, evidencing the presence of the DAC. Namely, the azacrown ether benzene ring is revealed with the ternary carbon C12 at 160 ppm (see ^13^C solution NMR spectrum for DAC in Figure 2). The strong signal around 117 ppm might correspond to some unreacted C7 allyl carbon together with contribution from the C13 and C14 of the DAC (see ^13^C solution NMR spectrum for DAC in Figure 2). New CH_2_ signals can be observed at 14.4 and 31.2 ppm (C8′) and might correspond to two different CH_2_ chemical environments [31]. Also striking is the appearance of a chemical shift at 181.8 ppm. It might be associated with a double imine bond (N=C) forming between chitosan amine and the allyl C=C bond of DAC. Such observations have been reported by other authors [25,26,32]. Finally, the C1 and C4 signals of chitosan shifted to 98.0 ppm and 86 ppm, respectively (these could be due to the changing chemical environment after reaction), while the C2 shoulder of chitosan is obscured within the broad C6 signal at 61.4 ppm.

#### 2.2.2. Model Studies to Verify the Chemical Reaction: Hydroamination of Azacrown Ether (DAC) by Glucosamine

**Scheme 3:**
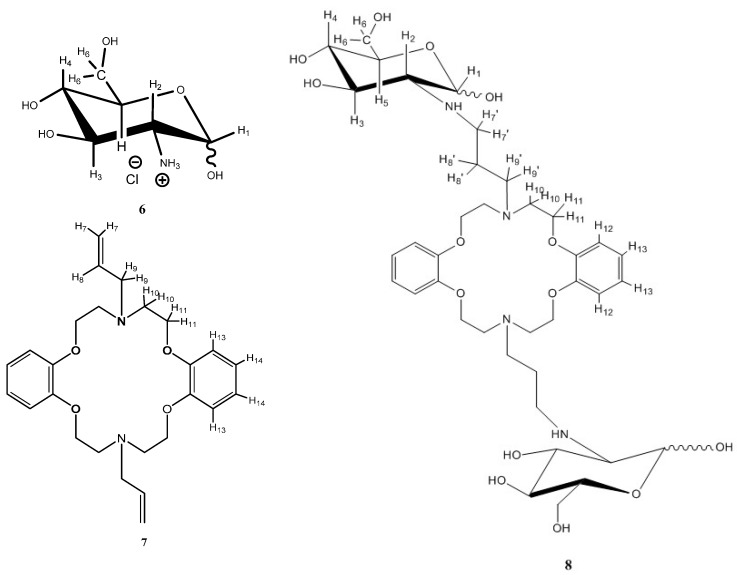
Schematic chemical structure of Glucosamine hydrochloride (**6**), DAC molecule (**7**) and Glucosamine/DAC complex (**8**).

To further study the feasibility of the reaction between the amine groups of Ch and the allyl groups of azacrown ether, D-Glucosamine hydrochloride (GlcN) was used as a model compound and reacted with the DAC (see molecules **6**–**8**, Scheme 3). The solution carbon-NMR spectra (Figure 2) of the reaction product reveals the disappearance of R–CH=CH_2_ unsaturated allyl carbons [29] at δ = 136.3 and 116.6 ppm, in agreement with its expected reaction with the amino group. The linkage subsequently becomes –CH_2_– aliphatic, with new signals for carbons 7′, 8′ and 9′ located at δ = 65.9, 25.2 and 56.6 ppm, respectively. This is clear evidence that a reaction between GlcN amine and R–CH=CH_2_ of DAC occurs, thus confirming the interpretation of the ^13^C solid NMR spectra of Ch-DAC. All other signals for GlcN and DAC remained unchanged and were assigned per the heteronuclear single quantum coherence-NMR spectra represented in the Supplementary data (Figure S2).

**Figure 3:**
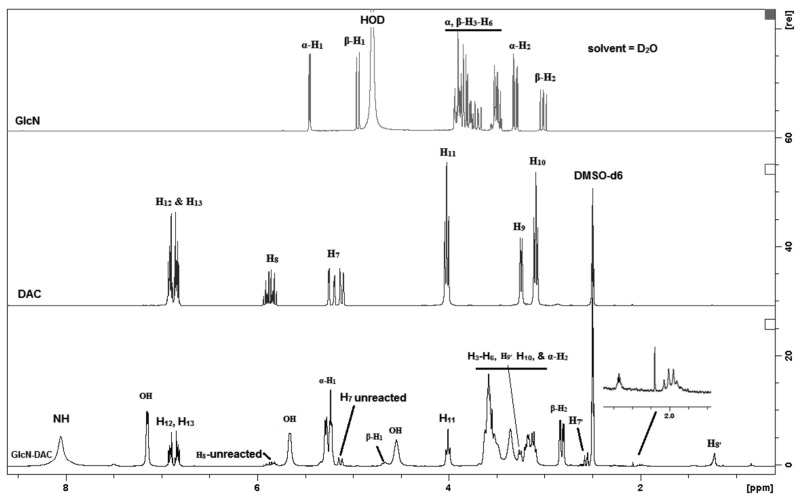
Proton-NMR spectra of glucosamine (GlcN), azacrown ether (DAC) and glucosamine-azacrown ether complex (GlcN-DAC).

The proton-NMR spectra of the reactants and the product are also shown in Figure 3. It reveals some important information, even though many proton signals overlap in the region 3–4 ppm. The appearance of new peaks 1.7, 2.2 (H8′), 2.58 (H7′) and 3.1 (H9′) ppm confirms the formation of aliphatic CH_2_ bonds. Also, there is an NH signal at 8.1 ppm associated with the new C–N (H) bond. The appearance of a weak original signal for DAC at 5.2 and 5.8 ppm associated with the R–CH=CH_2_ unsaturated allyl branch confirms the presence of residual, unreacted allyl groups in the bifunctional DAC. In other words, some amount of DAC might have reacted to chitosan with only one allyl arm, thus forming grafts onto the chitosan backbone rather than acting as crosslinker (Scheme 1). Furthermore, it is interesting to note that in the model study with glucosamine, no downfield carbon signal is further observed around 181 ppm, as previously observed in the solid-state NMR of the DAC-chitosan product. This confirms that the imine resonance proposed in the crosslinked system is associated with the acetylated units of chitosan. From these NMR data, it is concluded overall that the azacrown ether does react through its allyl group with the amine of the Ch and that this reaction might not be complete, leaving residual allyl functionalities.

#### 2.2.3. Fourier Transform Infrared (FTIR) Analysis

**Figure 4:**
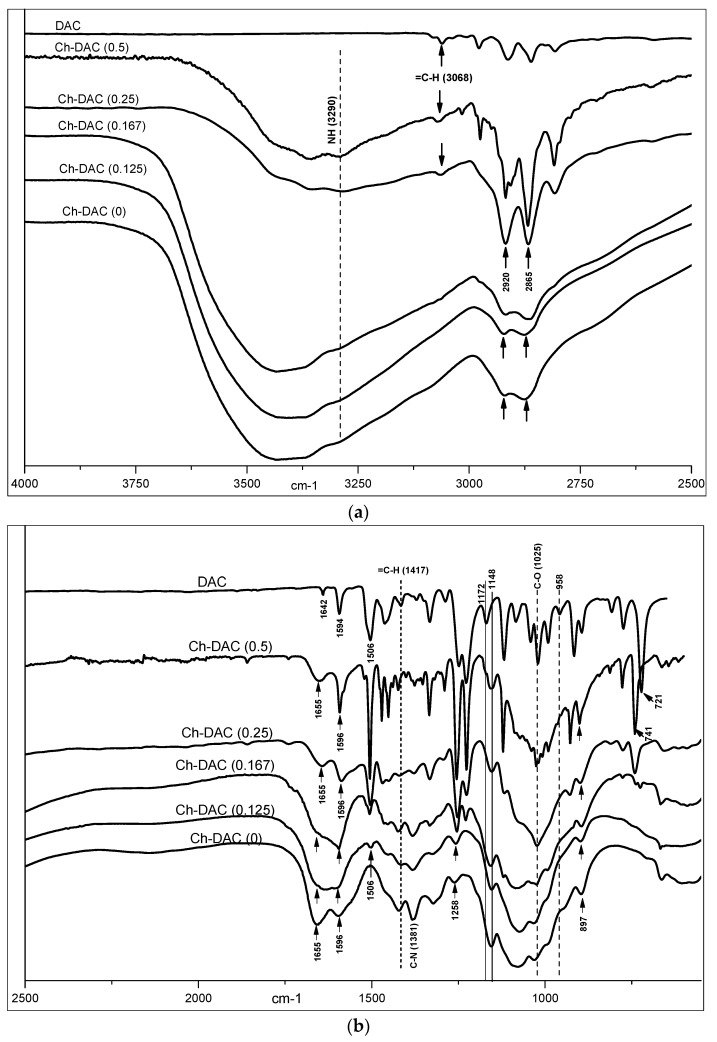
(**a**,**b**) Fourier transform infrared (FTIR) spectra of chitosan, Ch-DAC derivatives in various Ch/DAC molar ratios and DAC.

In Figure 4a,b, the comparison of the individual spectra of the neat chitosan, DAC and Ch-DAC films confirms the presence of azacrown ether in the films with the characteristics aromatic benzene ring vibrations at 1506 cm^−1^ and aromatic ether at 1258 cm^−1^ [33]. Likewise, chemical reaction between allyl and amine is suggested by the disappearance in the films of the stretching vibrations of the unsaturated C=C bond at 1642 cm^−1^ and the bending vibration at 1417, 958 and 721 cm^−1^ regions [29]. Simultaneously, the asymmetric and symmetric aliphatic chain signals of the newly formed NH–CH_2_CH_2_CH_2_–N bridge and the N–CH_2_CH_2_–O fragments of the crown cavity (δ = 2976–2807 cm^−^^1^) appear to intensify in the Ch-DAC signals. From the FTIR spectra of neat Ch film, it can be found that the distinctive absorption bands appear at 3446 cm^−1^ (–OH), 1655 cm^−1^ (amide I), 1596 cm^−1^ (–NH_2_ bending), and 1383 cm^−1^ (amide III) [34]. The amide I and the free amine bands overlap significantly with the stretching frequency of the crown ether benzene at 1594 cm^−1^, making it difficult to distinguish them around this region. Whereas the 1594-peak for DAC continues to increase in intensity, the 1655-peak for Ch reduces as DAC loading increases. It was also noted that the characteristic signature peak for β-glycosidic bond at 897 cm^−1^ is consistent, which indicates that the Ch chain backbone remains unchanged after the reaction.

#### 2.2.4. Stoichiometric Control of the Composition

**Figure 5:**
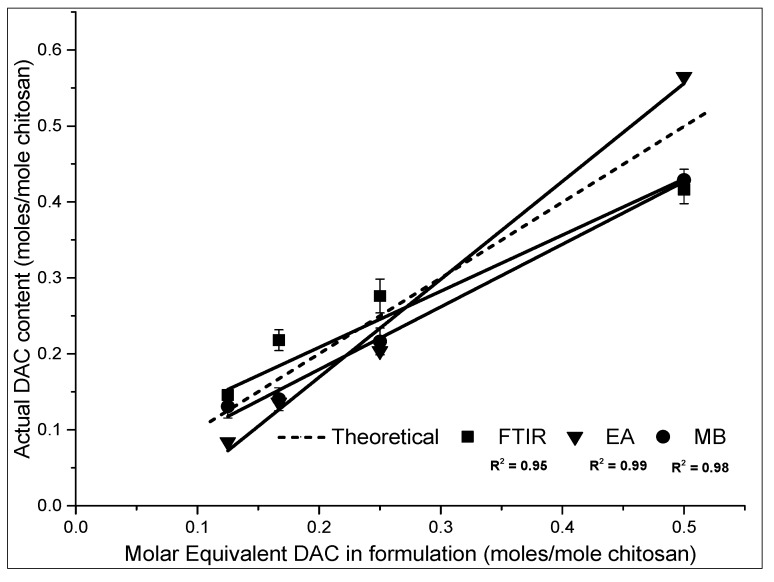
Linear relationship comparison between the molar equivalent DAC and the DAC content of the films measured from FTIR, EA and MB.

***Compositional Analysis by FTIR:*** From the infrared spectra, the peak area ratio between the 1506 cm^−1^ signal of benzene ring and the non-overlapping peak area at 1148 cm^−1^ for chitosan, A_1506_/A_1148_, was used to estimate the DAC content of the films according to Equation (1) [35]. All spectra were first normalized at 1025 cm^−1^ to absorbance 1. Figure 5 shows a positive linear relationship between the molar content of DAC used in the reaction and the actual DAC content. A comparison was made to the results from elemental analysis and mass balance.

(1) $$DAC\;Content=\frac{\int \frac{1506}{1025}/\int \frac{1148}{1025}}{\int \frac{1506}{1025}}$$

***Compositional analysis by EA and mass balance (MB)***: DAC content was also determined by elemental analysis and mass balance; and these experimental values were compared with theoretical compositions assuming complete reaction. For elemental analysis (EA) (Table S1 in the Supplementary data), a molecular formula unit of C_6_H_12_N_0.95_O_4.4_ was established from the results to represent the starting Ch while for DAC it was C_26_H_34_N_2_O_4_. Assuming 100% conversion, a model curve to represent several Ch-DAC formulations was computed using several theoretical DAC mole fractions as follows (Equation (2)):

(2) $$\left(C_{6}H_{12}N_{0.95}O_{4.4}\right)+\left\{\mathrm{DAC}\;\mathrm{mole}\;\mathrm{fraction}\times \left(C_{26}H_{34}N_{2}O_{4}\right)\right\}=\left(C_{n}H_{m}N_{x}O_{y}\right)$$

where $C_{n}H_{m}N_{x}O_{y}$ is the impirical formula to be determined from the EA results. Experimental results from elemental analysis for Ch-DAC films were fitted into the resulting curve equation to find actual corresponding DAC content in the samples. A linear relationship exists in all three methods for all chitosan/DAC mole fractions. Accordingly, the average DAC content was found to increase with reagent concentration in a near-stoichiometric proportion. From the linear regression analysis, it may be concluded that the results from EA were most consistent. However, at highest DAC content (DAC) = 0.5 the calculated results show more than theoretical value (0.56) while at the lowest DAC content (0.125) the calculated value is much lower (0.084). On the other hand, the calculated values for FTIR and mass balance (MB) tend to agree both at the lowest and at the highest ratios. It was therefore concluded that results from mass balance were more representative for the DAC content of the films.

### 2.3. Gel Content

**Figure 6:**
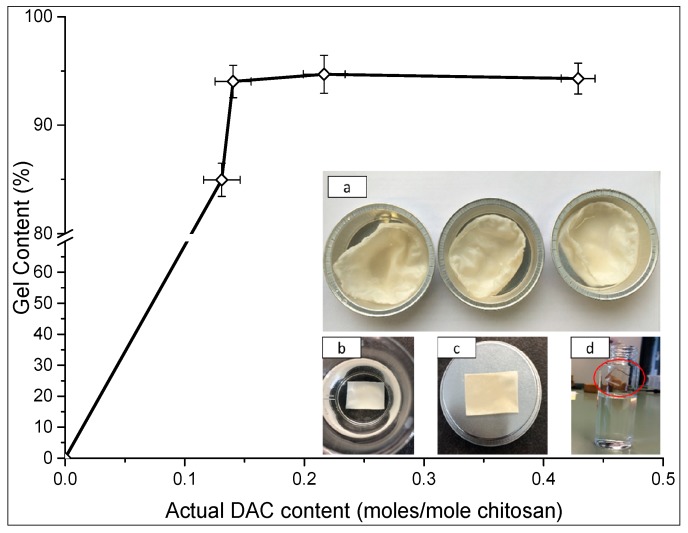
Gel content of the films with increasing DAC content. Inset: Photographs showing (**a**) wet films after Soxhlet extraction; (**b**) an insoluble film in 2% (*v*/*v*) acetic acid solution; (**c**) the wet film after solubility test; and (**d**) uncrosslinked chitosan film before complete dissolution occurred.

To confirm that Ch was effectively crosslinked by the azacrown ether, viz, that a network formed, the gel content of the films in acidic solutions was measured (Figure 6). As expected, neat Ch was readily soluble in acetic acid and HCl solutions; in contrast, the grafted hydrogel films were insoluble, albeit swollen in water, 0.1 M HCl, and 2% *v*/*v* acetic acid (see inset photographs in Figure 6). Gel content determination of the azacrown ether-modified films via Soxhlet extraction with 2% acetic acid solution indicated rapid network formation with increasing DAC content. Beyond the Ch:DAC molar ration of 1:0.125, which corresponded to a 15% DAC content in the final film, a maximum gel content of 95% was achieved, confirming effective crosslinking with a minimal chitosan sol fraction. Additionally, solubility tests revealed that all the films were insoluble in common organic solvents, viz, dimethylformamide, dimethyl sulfoxide, tetrahydrofuran, chloroform, dichloromethane, acetone and methanol.

### 2.4. Degree of Substitution

Incorporation of azacrown ethers into chitosan to act as a crosslinking agent has not been fully investigated. In particular, there are no reports in the literature of network density for crown ether crosslinked samples. Several factors may account for this information gap. One factor is that, for instance, to use the swelling data in calculating the Flory polymer-solvent interaction parameter, the molar volume fraction of solvent and polymer must be determined accurately. Lack of suitable swelling solvents other than water/acetic acid and the fact that at low crosslinking density the hydrogels swell extensively may also lead to inaccurate determination of the necessary parameters [36,37]. The microanalytical data (C, N) obtained from elemental analysis was therefore used to find out the degree of substitution (DS) of Ch-DAC. From Table S1 in the supplementary data, as already been mentioned in Section 2.2.4, C/N ratios of Ch and Ch-DAC are calculated as 5.40, Ch-DAC (0); 6.37, Ch-DAC (0.125; 7.81, Ch-DAC (0.167); 8.34, Ch-DAC (0.25) and 10.02, Ch-DAC (0.5). The DS of the derivatives to –NH_2_– group on chitosan is calculated by the Equation (3) [38].

(3) $$DS=\frac{a\left(C/N\right)m-{\left(C/N\right)}_{o}}{n}$$

where (C/N)m is the C/N ration of modified chitosan, Ch-DAC, ${\left(C/N\right)}_{o}$ is the C/N ratio of original chitosan (Ch) and “a” and “n” are the number of nitrogen and carbon introduced after DAC modification, respectively. The DS calculated for Ch-DAC was found as follows: Ch-DAC(0) = 0.0; Ch-DAC(0.125) = 0.28; Ch-DAC(0.167) = 0.39; Ch-DAC(0.25) = 0.43 and Ch-DAC(0.5) = 0.56. These results clearly confirm that as the molar equivalent DAC in formulation increased, so did the DAC content grafted.

### 2.5. Microstructural Characterization of the Azacrown Ether/Chitosan Films 

#### 2.5.1. Scanning Electron Microscopy (SEM) Images of the Synthesized Films

**Figure 7:**
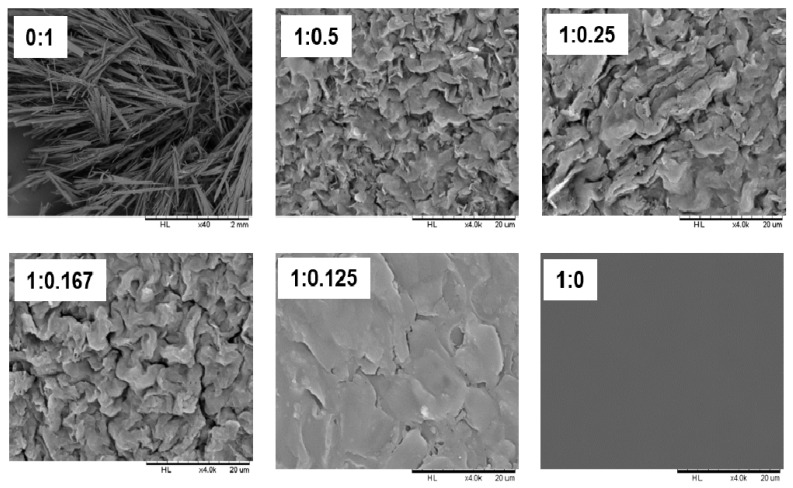
Scanning electron microscopy (SEM) micrographs of samples showing changes in surface texture as a function of DAC content (image magnification is 20 µm). The lower the DAC ratio, the more homogeneous film-like forming abilities. The first image shows pure DAC fibrous crystalline structure with image magnification 2 mm.

The physical features of the film surfaces were observed by scanning electron microscopy (Figure 7). These images give the overview and morphology of the films at 20 μm magnification. The neat chitosan films exhibit a smooth surface, while the crystalline structure of DAC is revealed by needle-like shape (×2 mm). As the concentration of DAC increases from 0 to 0.5 moles per mole of chitosan, the sample surface becomes rougher and porosity develops. The pores become denser and more uniform, which leads to production of microporous hydrogels. The DAC-treated films do not show any agglomerates or non-uniform surfaces. This confirms that the films exhibit a single phase homogeneous morphology on this scale of observation.

#### 2.5.2. Crystalline Microstructure

**Figure 8:**
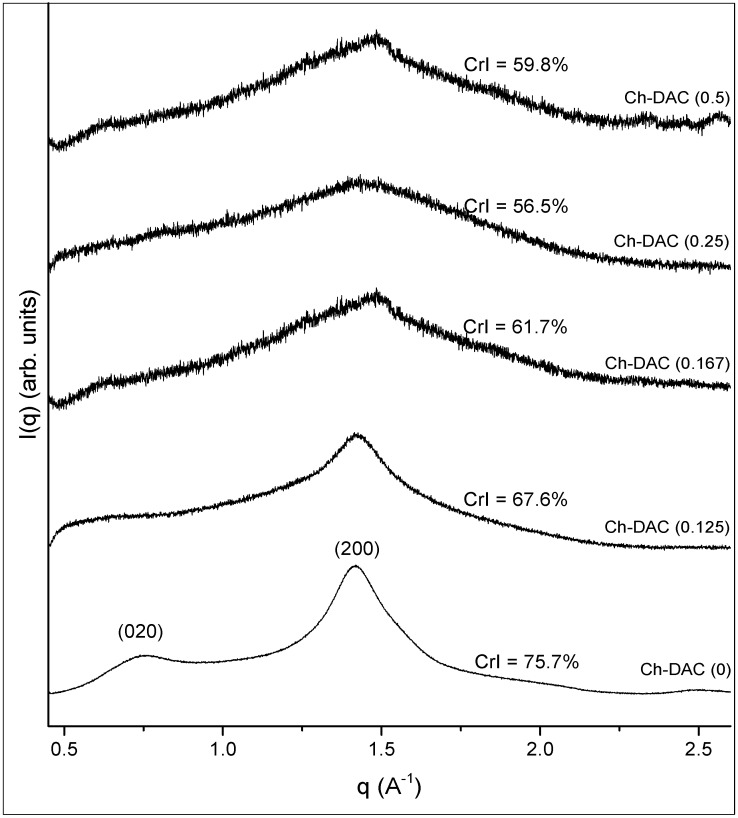
Wide-angle X-ray scattering (WAXS) patterns of chitosan (Ch) and azacrown ether-crosslinked chitosan films illustrating the impact of increasing DAC molar ratio on the Ch crystallinity index (CrI).

**Figure 9:**
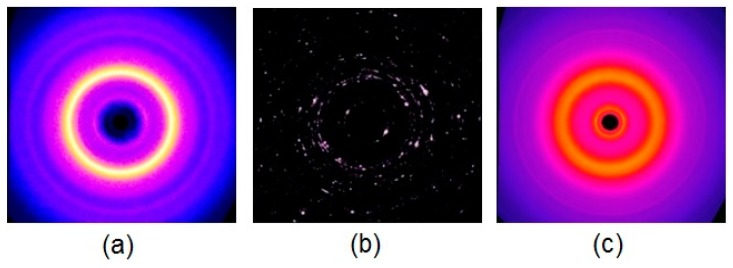
2D Synchrotron-WAXS images of (**a**) chitosan; (**b**) DAC and (**c**) azacrown ether-crosslinked chitosan derivative of Ch-DAC (0.25).

The effect of DAC grafting on crystallinity of the Ch-based films was studied by X-ray diffraction, using both conventional and synchrotron X-ray sources (Figure 8 and Figure 9, respectively). The diffractograms are consistent with those reported in the literature [39]. When comparing the azacrown ether-crosslinked Ch materials to that of neat chitosan, it is clear that increasing DAC content leads to a gradual crystallinity decrease (Figure 8) as previously reported by Ding et al. [23]. This indicated that the reaction of the crown ether with the amine groups of Ch destroys the H-bond arrangement present in the crystalline packing structure of chitosan [40].

**Figure 10:**
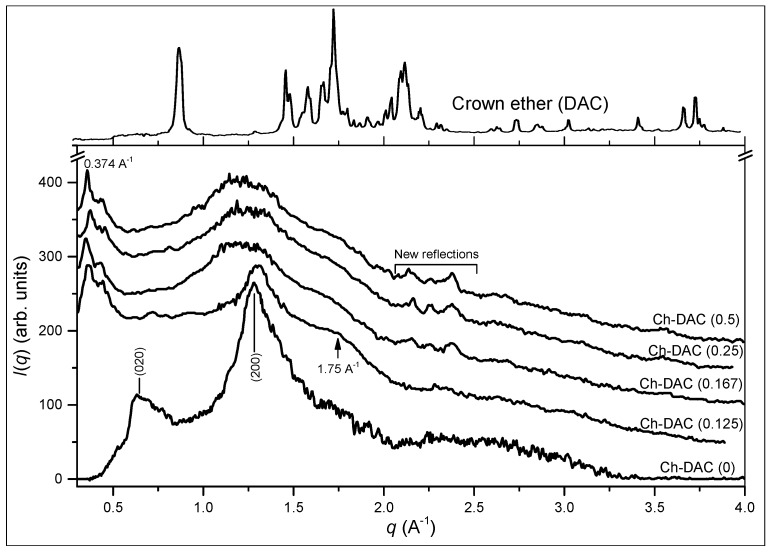
The radial averages of the 2D synchrotron-WAXS images. The radial averages for the pure azacrown ether compound are shown on top.

The synchrotron X-ray diffraction analysis allowed better characterization of the crystalline properties of the obtained materials. The Figure 9 shows 2D synchrotron wide-angle X-ray spectroscopy (WAXS) images of the chitosan, the azacrown ether compound and the azacrown ether-derivatized chitosan materials. New reflections appeared in the diffractogram after grafting of DAC on the chitosan, and some reflections of Ch were difficult to be distinguished (e.g., the (020)) in the Ch/DAC patterns, even more so when the DAC ratio increased (Figure 10). The synchrotron image of the pure azacrown ether compound shows a diffraction pattern of a textured powder sample (Figure 9b). Because not all crystallite orientations are equally represented in the textured sample, the reflections are concentrated in certain regions of reciprocal space and the pattern shows diffraction spots. This result can be compared with the DAC morphology studies performed by scanning electron microscopy (Figure 7). In effect, the SEM micrograph reveals a needle-like morphology for the DAC compound, with very elongated objects, whose synchrotron pattern shows the achievement of a practically 100% crystalline product (see also the radial averages in Figure 10). With this needle-like morphology and highly crystalline elongated objects, reflections of the corresponding single-crystal pattern can be separated in the synchrotron pattern from the obtained DAC textured sample (Figure 9), as its pattern generated a single-crystal-like data. The radial averages of the 2D synchrotron WAXS (s-WAXS) images show that the pattern of the starting Ch film has a main signal at q ~ 1.31 Å^−1^ (200) and a minor one at q ~ 0.66 Å^−1^ (020) corresponding both to reflections of hydrated allomorph of chitosan [41]. In contrast, the new Ch/DAC derivatives showed a significant reduction of the (020) reflection and new reflections at q = 0.374, 1.75, 2.15 and 2.38 Å^−1^. The WAXS results confirm that addition of azacrown leads to decrease of crystallinity of Ch, as expected. It is worth noticing that there are no peaks exactly at the position of the pure DAC diffraction reflections, confirming that the films do not possess a pure crystalline phase of DAC.

## 3. Materials and Methods

### 3.1. Starting Materials

The commercial chitosan, Ch, from shrimp shell chitin, was supplied by Sigma-Aldrich (Munich, Germany, Lot No. BCBJ172V) as pale-white powder with a moisture content ~7% as found by thermogravimetric analysis (TGA) measurements. Prior to its use, the starting chitosan was characterized through various methods to ascertain its properties.

***Molecular weight*.** Weight- and number-average molecular weights of the starting chitosan (Ch) were characterized by size exclusion chromatography (SEC) coupled to multiangle laser light scattering (MALLS) detection, following the procedure used by Osorio-Madrazo et al. [42]. Chitosan solutions at 0.1% (*w*/*v*) were prepared in an AcOH (0.2 M)/AcONH_4_ (0.15 M) (pH 4.5) buffer, used as eluent, then filtered through 0.45 μm pore-size membranes (Millipore, Darmstadt, Germany). The chromatographic equipment was composed of an IsoChrom LC pump (Spectra-Physics, San Jose, CA, USA) connected to a Protein Pack 200 SW (WATERS) column and a polymethacrylate-based TSKgel PW-type column (G6000 PWXL, Tosoh, Stuttgart, Germany). A multiangle laser light scattering (MALLS) detector DAWN DSP (Wyatt Technology, Toulouse, France) operating at 664.0 nm was coupled on line to a WATERS 410 differential refractometer (Milford, MA, USA).

***Degree of acetylation (DA)*.** The degree of acetylation was determined by ^1^H NMR. A total of 10 mg of Ch was dissolved in 1 mL of D_2_O acidified with 5 μL of concentrated HCl (12 M). NMR spectra were recorded on Avance DPX 300 MHz nuclear magnetic resonance spectrometer (Bruker, Wien, Austria) (300 MHz for ^1^H) at 70 °C. The DA was calculated, as proposed by Hirai et al. [43] from the ratio of the methyl proton signal of the (1→4)-2-acetamido-2-deoxy-β-d-glucan residues to the whole H2 to H6 proton signals.

***Crystallinity and X-ray scattering measurements.*** Chitosan flakes were analyzed by X-ray diffraction for estimating CrI. Wide-angle X-ray scattering (WAXS) patterns were recorded in reflection mode with a diffractometer (STOE, Darmstadt, Germany) operating at 40 kV and 30 mA with the Cu Kα1 radiation. The diffraction angle 2*θ* varied between 5° and 80° by steps of 0.04°. CrI was determined from the ratio of the crystalline contribution estimated from the crystalline peaks to the total area of the diffractograms, as previously reported by Osorio-Madrazo et al. [42].

The azacrown ether was synthesized and characterized in the laboratory according Toeri and Laborie [29]. It was in form of white fibrous crystals, with a molecular weight of 438.6 g/mol. All other chemicals were of analytical grade and used without further purification.

### 3.2. Preparation of Azacrown Ether/Chitosan Hydrogel Films

All chitosan/azacrown ether films used in this study were prepared by solvent casting onto glass plates from reaction mixtures formulated to contain a total solid content of 2 wt %. Five different film formulations were prepared by mixing chitosan (DA 23%) and DAC in molar ratios: Ch:DAC = 1:0, 1:0.125, 1:0.167, 1:0.25 and 1:0.5. In a typical preparation procedure, purified Ch (1.0 g) was first dissolved in deionized water containing 2% (*v*/*v*) acetic acid and heated to 40 °C. A total of 5 mL of iron(III) chloride hexahydrate solution was added and the mixture was stirred for 15 min under nitrogen flow. A required amount of N,N-diallyl-dibenzo-18-crown-6 dissolved in ethanol was slowly dropped into the mixture so that at the end a 2 wt % solid content was achieved. The resulting mixture was then refluxed at 40 °C under continuous stirring for 24 h, followed by addition of 5 mL H_2_O_2_ solution. After the reaction was completed, 10 mL aliquots were transferred onto cylindrical glass Petri dishes (50 mm W *×* 10 mm H, Pyrex, Corning, NY, USA) and dried in air for two days followed by oven drying at 60 °C for 24 h. The films were peeled off the glass plates and neutralized by immersion in a NaHCO_3_ solution. Then, they were washed to pH 7 to give pale white products. The wet films were extracted with ethanol in a Soxhlet extractor for 4 h to eliminate any unreacted crown ether before drying. Five formulations designated as Ch-DAC (0), Ch-DAC (0.125), Ch-DAC (0.167), Ch-DAC (0.25) and Ch-DAC (0.5) based on increasing the DAC mole ratio to one mole of Ch, were manufactured.

### 3.3. Model Studies to Verify the Chemical Reaction: Hydroamination of DAC by Glucosamine (GlcN-DAC)

To conveniently study the hydroamination reaction of the allyl group of DAC by chitosan, a model reaction was conducted by mixing glucosamine (GlcN) with DAC under similar reaction conditions to the Ch-DAC reaction. The product GlcN-DAC was readily soluble in dimethyl sulfoxide and monitored with solution NMR.

### 3.4. Chemical Structure Analysis

***Fourier transform infrared spectroscopy.*** The Fourier transform infrared (FTIR) spectra of the films were recorded in attenuated total reflection (ATR) mode on a FTIR spectrometer 65 (Perkin Elmer, Waltham, MA, USA) using 32 scans at a resolution of 2 cm^−1^. After measurements, the spectra were ART corrected. All samples were dried at 60 °C for 24 h prior to measurements. 

***NMR spectroscopy.*** Proton-, carbon- and 2D- solution NMR was conducted on an Avance DPX 300 MHz nuclear magnetic resonance spectrometer. Ch was prepared by dissolving 10 mg/mL for ^1^H-NMR and 20 mg/mL for ^13^C-NMR in D_2_O/HCl (100/1 *v*/*v*) and measured at 70 °C. Glucosamine 10 mg/mL was dissolved in D_2_O for both ^1^H- and ^13^C-NMR. Glucosamine/azacrown complex was dissolved in DMSO-d6 (20 mg/mL) for both proton and carbon NMR, while azacrown ether was measured in aceton-d6. 

***Elemental analysis.*** EA was done on dried samples using a Vario elemental analyzer (Elementar Analysensysteme GmbH, Hanau, Germany).

### 3.5. Gel Content

To evaluate the success of network formation in Ch films, the gel content of the films was determined via Soxhlet extraction. While uncrosslinked chitosan will dissolve in hot dilute acetic acid, crosslinked portions of chitosan will not. Whole film samples from each composition were dried, weighed, and extracted with 2% acetic acid for 24 h, as per ASTM 2765 [44]. The samples were then removed, dried again, and reweighed. The resulting extracted film specimens provide information about the percent fraction of the Ch film successfully crosslinked. The gel content was calculated using the following Equation (5).

(4) $$extract\;\left(\%\right)=\frac{weight\;lost\;during\;extraction}{weight\;of\;original\;specimen}\times 100$$

(5) gel content=(100−extract) %

Further tests were performed by testing the solubility of films in each of distilled water, 0.1 M HCl and 2% *v*/*v* AcOH. Film strips measuring ~2 × 2 cm^2^ were immersed into the solutions for a period of 24 h with stirring in a shaker at 150 rpm. The films were then withdrawn from the solutions, dried and weighed.

### 3.6. Microstructural Characterization of the Azacrown Ether/Chitosan Films

***Microstructure studies by scanning electron microscopy (SEM).*** The physical structure of the films was observed with a Hitachi TM3000 tabletop scanning electron microscope operating at an accelerating voltage of 15 kV. The dried film samples were mounted on a metal stub and sputtered with gold to make the sample conductive.

***Synchrotron and laboratory source WAXS measurements.*** X-ray patterns of Ch, crown ether, and chitosan-crown ether films were recorded both with a conventional laboratory source as described in Section 2.1 for the starting chitosan; and with a synchrotron X-ray source. The synchrotron WAXS patterns in transmission mode were recorded at the BM02/D2AM beamline at the European Synchrotron Radiation Facility (ESRF), Grenoble, France (photon energy: 16 keV, λ = 0.7749 Å). Data were collected using a charge coupled detector camera; CCD detector; (Ropper Scientific, Vianen, Netherlands). The contribution of the empty cell was subtracted from the scattered data. Silver behenate was used as q-range calibration standard. 

## 4. Conclusions

Chemical modification of chitosan with N,N-diallyl-7,16-diaza-1,4,10,13-tetraoxa-dibenzo-18-crown-6 can be accomplished to produce crosslinked films with up to 50% DAC content in a controllable stoichiometric manner. Incorporating the azacrown ether into chitosan backbone as a crosslinking agent provided the opportunity to stabilize chitosan and thus could be applied in acidic pH solutions. The influence of DAC as a cross-linking agent on the properties of chitosan films was also investigated. With a DAC content above 15%, crosslinking is sufficient to produce stable, acid and organic solvent-insoluble films, with a high gel content of 95%. In this case, the chitosan X-ray and SEM data analyses showed that the derivatives exist as homogenous one-phase materials, in which the chitosan crystalline morphology is slightly reduced and in which DAC acts as crosslinker of chitosan. FTIR, EA and mass balance analyses confirmed that the composition of azacrown ether-crosslinked Ch could be controlled via stoichiometry of the reacting species. Considering the fact that azacrown ethers are also good chelators with varying sets of donor atoms having different meta ion affinities, the resulting Ch/azacrown copolymer would be expected to have increased reactivity for effective recovery of various metal ions from wastewater, enhancing its selectivity for specific metal ions, or increasing the adsorption capacity. Heavy metal adsorption capacity of this family of azocrown-ether- crosslinked Ch will be reported in a following publication.

## Supplementary Materials

The following are available online at www.mdpi.com/1996-1944/10/4/400/s1. Figure S1: Proton NMR spectrum of chitosan with DA = 23% used in this study, Figure S2: Size Exclusion chromatography/multiangle scattering (SEC/MALLS) spectrum obtained to calculate the weight- and number-average molecular weight and polydispersity index, Figure S3: The HSQC spectrum for assigning the carbon signals with their corresponding protons. Table S1: Elemental Analysis data for Ch-DAC hydrogel films.
