# Supplementary material for: Preparation and Chemical/Microstructural Characterization of Azacrown Ether-Crosslinked Chitosan Films
Source: Materials (Basel). 2017 Apr 11;10(4):400. doi: 10.3390/ma10040400 (PMC5506952; doi:10.3390/ma10040400)
Supplement: Supplementary file 1 [file materials-10-00400-s001.pdf]

Supplementary Materials: Preparation and Chemical/Microstructural Characterization of Azacrown Ether-Crosslinked Chitosan Films

Julius Toeri, Anayancy Osorio-Madrazo and Marie-Pierre Laborie

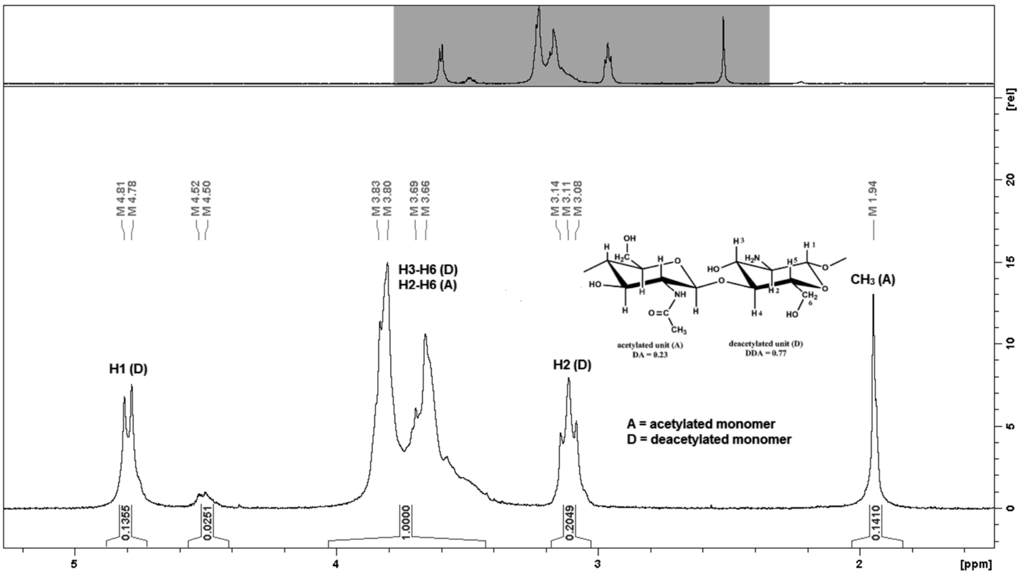

Figure S1. Proton NMR spectrum of chitosan with DA = 23% used in this study.

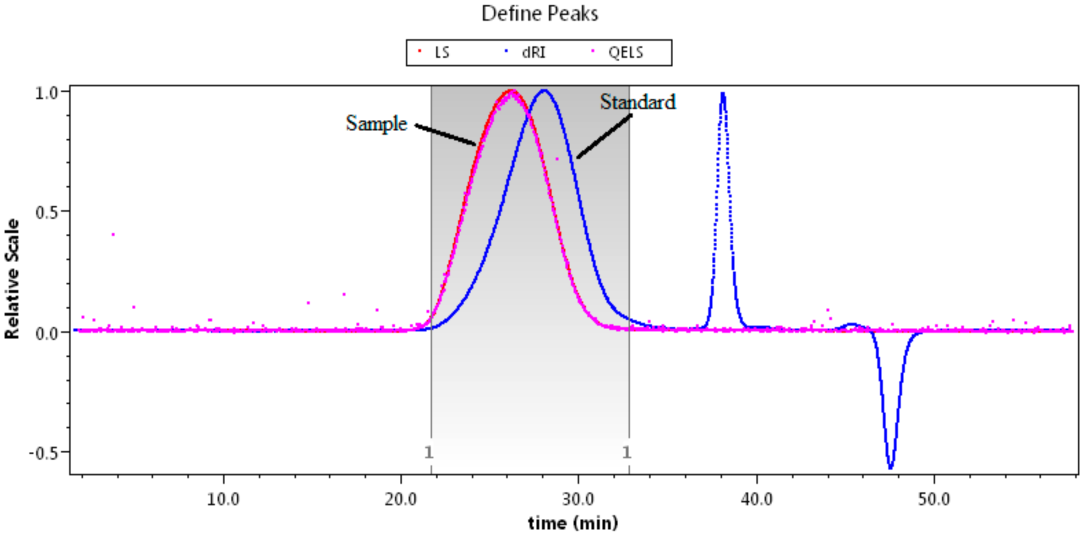

Figure S2. Size Exclusion chromatography/multiangle scattering (SEC/MALLS) spectrum obtained to calculate the weight- and number-average molecular weight and polydispersity index.

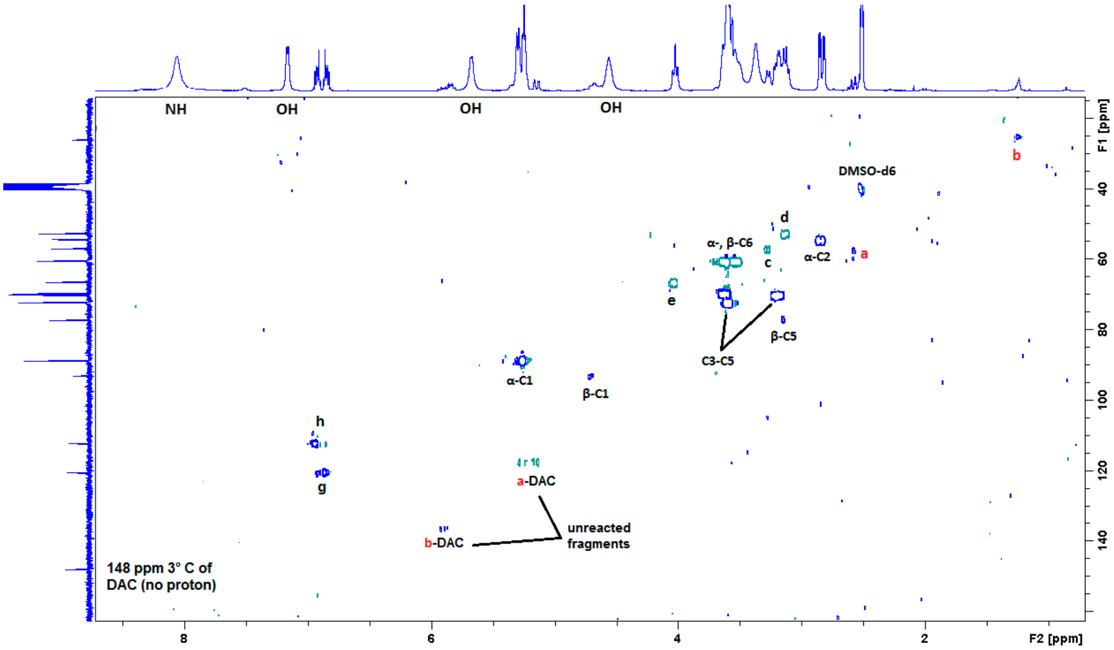

Figure S3. The HSQC spectrum for assigning the carbon signals with their corresponding protons.

Table S1. Elemental Analysis data for Ch-DAC hydrogel films.

Compound	[Ch]	[DAC]	Elemental analysis				
			C%	H%	N%	O%	C/N
Ch-DAC (0)	1.0	0	42.78	7.49	7.92	41.81	6.297
Ch-DAC (0.125)	1.0	0.125	44.51	7.12	8.15	40.22	6.372
Ch-DAC (0.167)	1.0	0.167	47.85	6.97	7.15	38.03	7.808
Ch-DAC (0.25)	1.0	0.25	51.34	7.08	7.18	34.4	8.342
Ch-DAC (0.5)	1.0	0.5	55.33	7.13	6.44	31.1	10.024
DAC	-	-	71.08	7.85	6.30	14.77	13.152

© 2017 by the authors. Submitted for possible open access publication under the terms and conditions of the Creative Commons Attribution (CC BY) license (http://creativecommons.org/licenses/by/4.0/).
